# Combining the Integrated-Change Model with Self-Determination Theory: Application in Physical Activity

**DOI:** 10.3390/ijerph18010028

**Published:** 2020-12-23

**Authors:** Kei Long Cheung, Sander Matthijs Eggers, Hein de Vries

**Affiliations:** 1 Health Behaviour Change Research Group, Department of Health Sciences, College of Health and Life Sciences, Brunel University London, London UB8 3PH, UK; 2 Central Bureau for Statistics (CBS), 6401CZ Heerlen, The Netherlands; sm.eggers@cbs.nl; 3 Department of Health Promotion, CAPHRI Care and Public Health Research Institute, Maastricht University, 6200MD Maastricht, The Netherlands; hein.devries@maastrichtuniversity.nl

**Keywords:** integration, physical activity, theories, Self-Determination Theory, the I-Change Model, Theory of Planned Behaviour

## Abstract

*Background*: Critically testing and integrating theoretical models can aid health promotion research and intervention planning. This study aimed to critically compare and integrate Self-Determination Theory (SDT) and Integrated-Change Model (ICM) for explaining physical activity behaviour. *Methods*: A dataset was used with Dutch adults, consisting of information on demographics and socio–cognitive and behavioural determinants. There were three measurements over a period of six months, with the baseline sample consisting of 1867 participants. Confirmatory factor analysis was conducted to assess the reliability of the items and their corresponding scales. To examine cognitive pathways, we applied Structural Equation Modelling (SEM). *Results*: For SDT, none of the pathways were significant but the model fit was decent (R^2^ = 0.20; RMSEA = 0.07; CFI = 0.91). For ICM, the model fit was similar (R^2^ = 0.19; RMSEA = 0.07; CFI = 0.73), with many significant pathways, as stipulated by the theory. The integration of STD and ICM constructs revealed similar explained behavioural variance (R^2^ = 21%), with no significantly different model fit. *Conclusion*: The integration of SDT and ICM added no value as a prediction model. However, the integrated model explains the underlying mechanism of STD constructs, as well as the determinants of attitude, social influences, and self-efficacy. In the context of intervention design, ICM or the integrated model seem most useful as it reveals the stages and pathways to behaviour change.

## 1. Introduction

In order to understand health behaviour and to plan health promotion, theoretical constructs and models are used. The amount of theoretical constructs and models used in health behaviour science is enormous [1]. The literature showed that at least 1700 constructs are used in 83 theories [2], which are not all distinct [3] or can be operationalized separately [4]. As many of these theories have considerable overlap, as several scholars have attempted to integrate various models and theories [3,5,6,7]. Progress in health psychology will be contingent on testing numerous theories [8] and finding alternative pathways [5].

Theories such as the Social Cognitive Theory (SCT) [9], the Health Belief Model (HBM) [10], the TransTheoretical Model (TTM) [11], and the Theory of Planned Behaviour (TPB) [12] have been shown to be successful health behaviour models [13]. These models emerged from an integration of constructs from of other theories. For instance, SCT, and later also SCT, TBP and TTM, added self-efficacy to their model. TTM also incorporated principles from various therapy systems [11]. This integration process will probably be an ongoing scientific process and is particularly relevant when new constructs have added value and lead to new hypotheses [5]. The integration of constructs can also lead to different models. The Health Action Process Approach (HAPA) [14] proposes risk perceptions, outcome expectations (attitude) and task self-efficacy as the factors directly influencing a person’s motivation and intention. The Integrated-Change Model (ICM) [5] postulates a more distal role for risk perceptions that operate in the pre-motivational phase together with cognizance (awareness of one’s own behaviour), knowledge and perceived cues [5,15]. Actually, these differences help to challenge researchers to identify more clearly the role of the various factors involved in understanding behaviour [5]. In this study, we explore the integration of the ICM and the Self-Determination Theory (SDT) [16].

The ICM offers a socio–cognitive ecological framework for understanding health behaviour. Albeit sharing similarities to TPB, there are several important differences. First, the ICM suggests the existence of different phases in the process of behaviour change, such as awareness, motivation, and an action phase. Awareness is determined by cognizance, knowledge, risk perceptions, and cues to action. These awareness determinants are related to behaviour, but their effects are mediated by the factors determining a persons’ motivation or orientation to engage in a particular behaviour, such as attitudes, social influences and self-efficacy [5,15]; which are similar to the three motivational determinants of TPB. Addressing the intention–behaviour gap in TPB, the ICM postulates factors that are related to the action phase: self-efficacy, action planning and plan enactment [17], a finding also postulated by the HAPA model [18] and in line with a meta-analysis by Rhodes and Dickau (2012) [19]. Then, contrary to TPB, the ICM is intended as a flexible framework acknowledging the impact of various distal factors, for instance, contextual factors such as parenting styles and practices [20], and information factors such as the framing of messages [21]. Third, the ICM acknowledges the existence of dual process models distinguishing implicit and explicit cognitions. For instance, implicit attitudes were found to strengthen the relationship between attitude and intention, and moderated the relationship between self-efficacy and physical activity [22], and targeting both explicit and implicit associations were shown to be effective in changing behaviour.

Whereas the ICM model postulates the existence of distal psychological factors and has also outlined the importance of some of these factors, less attention is paid to the importance of values and psychological needs, constructs also identified by, for instance, Bandura (1986) [9]. This theory focuses more on the quality of motivation and the (environmental) factors that predict this motivation. SDT, outlined by Deci and Ryan (2000) [16], specifically addresses the importance of internal needs which are universal, innate and psychological and include the need for competence (i.e., feeling effective), autonomy (i.e., feeling the origin of one’s own behaviour), and relatedness (i.e., feeling understood and cared for by others). Satisfying these fundamental needs leads to the adoption of health-promoting behaviours, and improved physical and mental health [23]. Moreover, the acknowledgement of these needs as key elements of motivation makes SDT quite distinct from social cognitive theories. SDT thus provides a different approach to motivation, by distinguishing the quality of motivation and distinguishing these along a continuum moving from extrinsic or controlled motivation to self-determined or intrinsic motivation, which are referred to as: amotivation; externally regulated; introjected regulation; identification; integrated regulation; and intrinsic [16]). When motivation is self-determined, the individual experiences a sense of personal choice and autonomy and feel their behaviour represent their true self. Self-determined motivation was shown to positively affect behavioural engagement [24]. Various studies have successfully applied SDT to understand various health behaviours in various settings [25].

Attempts have been made to compare and integrate the TPB and SDT [7,26]. This is interesting as SDT does not detail how motivational orientations are converted into intentions and behaviour, whereas the TPB does not describe in detail the determinants of their three core motivational factors [27]. The previous studies suggest that self-determined motivation indirectly influences intention via TPB factors. Luqman and colleagues (2018) [26] demonstrated in a cross-sectional study that the effects of autonomous and controlled motivation concerning social network discontinuation intentions were fully mediated by attitudes, subjective norms and perceived behavioural control, and appeared to have no direct effect on discontinuance intentions. However, the literature is inconclusive with regard to this relationship [7,28]. Although Luqman and colleagues [26] concluded that autonomous and controlled motivation served as determinants of attitudes, subjective norms and perceived behavioural control, they did not test a potential reversed model. However, the three TPB determinants of behaviour is potentially mediated by the SDT constructs for instance. Given their cross-sectional design, the opportunities to investigate the mechanism is limited. More research is therefore warranted to detail the specific place of the two SDT constructs in the mechanism of behaviour change. Additionally, although findings from cross-sectional research do suggest causal pathways between SDT and TPB [7], the conceptual overlap between the components of the two theories (e.g., competency and perceived behavioural control) is not clear [29]. Exploring an integrated model of these constructs is therefore relevant.

There is a need for examining the similarities and differences in content and operationalizations of constructs from theories [30,31,32]. As ICM offers a more detailed approach than TPB, by also outlining pre-motivational and post-motivational factors, it is thus relevant to investigate how to integrate concepts of SDT with ICM. With successful applications of both theories, ICM and SDT, in explaining physical activity behaviour [33,34,35], this study utilises physical activity as a case behaviour. This leads us to the aim of this study, which was two-fold. In order to compare SDT and ICM for explaining physical activity behaviour using a longitudinal design, this study aimed to: (1) assess the model fit of ICM and SDT; and (2) assess whether the autonomy constructs (i.e., amotivation, external regulation, introjected regulation, identified regulation, intrinsic motivation) of SDT have additional and unique explained variance in explaining physical activity behaviour above the ICM constructs and vice versa. This will aid theory for the future research and planning of health interventions.

## 2. Materials and Methods

### 2.1. Sample

Data were gathered among Dutch adults. The sample was drawn from an online survey panel at random. Participants were excluded if they were indicated to suffer from any physical disabilities that would not allow regular physical activity as recommended by the Dutch Standard Healthy Movement [36]. In addition, the participants were excluded if they, were active zero minutes a day, were excessively active (>5 × Z-scores), or if they were older than 75 years. The final baseline sample consisted of 1867 participants (18–75 years; mean = 47 years). About half the sample, 51% (N = 960), was male and about 28% had finished university or any other form of higher education. These values are similar to those of the general Dutch population [37].

### 2.2. Procedure

An online survey panel (i.e., Flycatcher) was used to collect the data. The survey panel has an extensive list of registered members and participants were invited by e-mail. The e-mail informed participants that the study comprised three measurements over a period of six months and that their confidentiality would be ensured. In total, 4978 invitations were sent, of which 2434 participants clicked the activation link and completed the first survey online (49% response rate).

Participants were neither subjected to procedures, required to follow rules of behaviour, nor did the study involve scientific medical research. Thus, the study did not fall under the scope of the WMO (Medical Research Involving Human Subjects Act) and ethical approval was not required. Participants were allowed to leave the panel at any point of the study.

### 2.3. Questionnaire

The baseline questionnaire consisted of relevant demographic variables and 127 questions regarding socio–cognitive and behavioural determinants. Items were piloted among a small group, which resulted in minor language amendments.

Demographics: gender, age, length, weight and highest completed educational level were inquired. In order to calculate the body mass index (BMI) of participants, length in m and weight in kg were used (BMI = weight in kg/(Length in m × Length in m). Educational level was categorized into ‘low’ (no education, elementary education, medium general secondary education, preparatory vocational school, or lower vocational school), ‘medium’ (higher general secondary education, preparatory academic education, or medium vocational school), and ‘high’ (high vocational school or university level).

Awareness factors: Items reflecting awareness factors were based on the ICM and aligned to ICM-derived questionnaires [34,38]. Knowledge was assessed by six items that reflected statements that were either true or false (e.g., ‘Moderate physical activity means for adults for example biking without increased heart rate’), measured on a 3-point scale (1 = ‘true’, 2 = ‘false’, 999 = ‘I don’t know’). All items were recoded into 1 = ‘answered correctly’ and 2 = ‘answered incorrectly’/‘I don’t know’ and combined into an index. Risk perception was assessed by four items on a 5-point Likert scale. As suggested in the I-Change model, the items assessed susceptibility and severity separately. Two items evaluated the susceptibility of mental/physical disabilities being associated with physical inactivity (α = 0.56; e.g., heart disease or depression) on a scale reaching from 1 = ‘My chance to develop a disease like this is very low’ to 5 = ‘My chance to develop a disease like this is very high’. Two items estimated the severity of mental/physical risk of getting these diseases (α = 0.69), on a scale reaching from 1 = ‘If I would develop a disease like that, it would be for me not severe’ to 5 = ‘If I would develop a disease like that, it would be for me very severe’. Cues to action were assessed by eight items on a 5-point Likert scale reaching from 1 = ‘No, definitely not’ to 5 = ‘Yes, definitely’. Participants were asked which cues would prompt them to engage in physical activity (α = 0.79; e.g., ‘Seeing myself in the mirror’). Cognizance was estimated by three items (e.g., ‘I am sufficiently physical active to keep my health in balance’) on a 5-point Likert scale reaching from 1 = ‘totally disagree’ to 5 = ‘totally agree’ (α = 0.90).

Socio–cognitive factors of the I-Change model: Items reflecting socio–cognitive factors were based on the motivational phase of the ICM and aligned to ICM-derived questionnaires [34,38]. Attitudes were assessed by 20 items, each starting with the sentence ‘If I move sufficient, than …’. Ten items were framed as pros of physical activity (α = 0.895), and ten were framed as cons of physical activity (α = 0.86). Answering options ranged from, for example, 1 = ‘I do not feel more attractive’ to 5 = ‘I feel much more attractive’. Social influence was assessed by eight items regarding norms and modelling of partner, family, friends and colleagues. Four items examined social norms, answering options ranged from 1 = ‘… think(s) that I do not move sufficiently’ to 5 = ‘… think(s) that I move sufficiently’. The other four items assessed social modelling (e.g., ‘My partner is sufficiently physical active’) on a 5-point Likert scale ranging from 1 = ‘I totally disagree’ to 5 = ‘I totally agree’. Additionally, the option ‘I have none’ was included for all eight items. All items were included separately into the analyses due to low Cronbach’s alphas. Self-efficacy was assessed by nine items following the stem ‘I find it difficult/easy to be sufficiently physical active if…’. Answering options ranged from 1 = ‘Very difficult’ to 5 = ‘Very easy’ on a 5-point Likert scale. Intention was assessed by three items (α = 0.91) regarding ‘having the plan do be sufficiently physical active within three months’, ‘being motivated to be sufficiently physical active within three months’, and ‘the change of being sufficiently physical active within three months’ on a 5-point Likert scale ranging from 1 = ‘No, definitely not’/‘Totally disagree’/‘Very low’ to 5 = ‘Yes, definitely’/‘Totally agree’/‘Very high’, respectively.

Motivation factors of Self-Determination Theory: The motivation factors of SDT for physical activity was assessed by the Behavioral Regulation and Exercise Questionnaire (BREQ-3) [39]; including four complementary items on integrated regulation [40]. This means that the Dutch BREQ-2 questionnaire was used [41], with additional items of the BREQ-3 being translated and piloted. However, we agree that it is recommended to validate the scale for use in future research as no formal validation studies regarding BREQ-3 exist. We have now detailed this in the methods by referencing the above in the methods and limitations of the discussion with added references. The 24 items measured amotivation (e.g., I don’t see why I should have to be sufficiently physical active; α = 0.83 ), external regulation (e.g., I am sufficiently physical active because other people say I should; α = 0.83), introjected regulation (e.g., I feel guilty when I am not sufficiently physical active; α = 0.81) identified regulation (e.g., It’s important to me to be sufficiently physical active regularly; α = 0.73), integrated regulation (e.g., I am sufficiently physical active because it is consistent with my life goals; α = 0.88) and intrinsic regulation (e.g., I am sufficiently physical active because it’s fun; α = 0.89). Answers were given on a 5-point Likert scale ranging from 1 = ‘Totally agree’ to 5 = ‘Totally disagree’.

Planning: Items reflecting planning were based on the action phase of the ICM and aligned to ICM-derived questionnaires [34,38]. Preparatory planning was assessed with eight items (e.g., ‘Within one months I will pick a specific starting date to be sufficiently physical active’) on a 5-point Likert scale from 1 = ‘Totally disagree’ to 5 = ‘Totally agree’.

Physical activity. The outcome variable was assessed with the International Physical Activity Questionnaire (IPAQ)—short last seven days self-administration format [42]. The IPAQ assessed the frequency (days per week) and the duration (minutes per day) of walking, moderate-intensity activities and vigorous intensity activities. The mets minutes represented the amount of energy expended when carrying out physical activity; a multiple of one’s estimated resting energy expenditure. The IPAQ items were derived from the Dutch version of the International Physical Activity Questionnaire, which was shown to be a reliable and reasonably valid physical activity measurement tool for the general adult population [43]. The calculation and translations were in line with previous studies, with reliability and validity shown in 12 countries including the Netherlands [42]. A total score of physical activity per day was calculated in accordance with the literature. The resulting scores were somewhat skewed. Forty-three percent of the sample reported ‘high activity’ (>3000 mets), 31% moderate activity (1500–3000 mets), 19% low activity (500–1500 mets) and 6% insufficient activity (0–500 mets). To be able to differentiate more optimally between high and low activity individuals and in order to improve the normality of the distribution, physical activity was recoded as follows: 0 = very low activity (0–1000 mets); 1 = low activity (1000–2500 mets); 2 = moderate activity (2500–5000 mets); 3 = high activity (>5000 mets).

### 2.4. Statistical Analyses

The preparation of the data and descriptive analyses were carried out using R v3.2.3. Confirmatory factor analysis was done in R to assess the reliability of the items and their corresponding scales. All items loaded significantly on their respective factors (all loadings *p* < 0.01).

To examine the cognitive pathways, we applied Structural Equation Modelling (SEM), available in the R package Lavaan [44]. In terms of modelling, the physical activity measured at follow-up was regressed on baseline cognitive factors (e.g., motivation, self-efficacy) which were arranged in accordance with their respective theory. All cognitive factors were allowed to correlate with each other.

Modification indices (MI > 25) were examined to identify the missing paths and the Comparative Fit Index (CFI) and Root Mean Square Error of Approximation (RMSEA) were used for determining model fit. For the CFI, values over 0.90 indicated acceptable fit and values over 0.95 indicated good fit [45]. For the RMSEA, which controls for sample size, values lower than 0.08 indicate acceptable fit and values under 0.06 indicate a good fit [45]. All presented coefficients are unstandardized and effects are considered significant when α < 0.05. The dataset used is available from the corresponding author on reasonable request.

## 3. Results

### 3.1. Descriptive Statistics

The sample consisted of 1867 participants. Their average age was 47 years (SD = 16.2) and 26% of the sample was over 60 years old. About half the sample was male (51%; N = 960), and 28% had finished university or other forms of higher education. Scores that use the regular IPAQ categorization showed that 44% of the sample engaged in high physical activity, 31% in medium physical activity, 19% was in low physical activity, while 6% was not active at all. Since these scores are highly skewed and previous research has shown that the IPAQ can overestimate physical activity [46], it was decided to re-categorize the scores. Using normally distributed cut-offs of 5000, 2500 and 1000 mets, the results showed that 24% of the sample engaged in high physical activity, 30% was in medium physical activity, 32% was in low physical activity, and 14% was not active at all.

### 3.2. Model Fit of ICM and SDT

**Figure 1:**
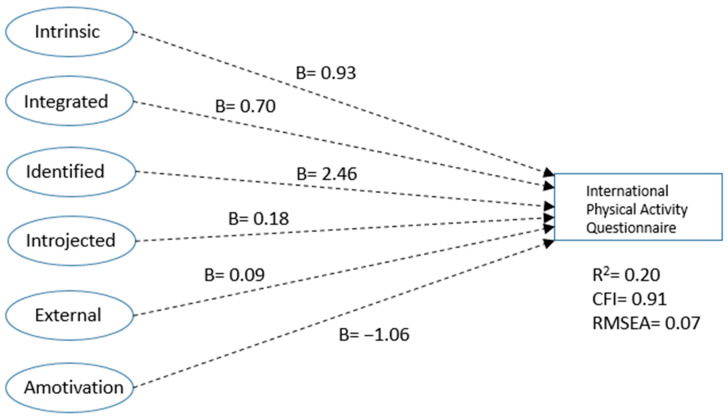
Unstandardized regression coefficients for Self-Determination Theory (SDT).

First, we assessed whether the basic SDT model would fit the data. IPAQ scores at 3-month follow-up were regressed on intrinsic, integrated, identified, introjected, external, and amotivation factors. None of the pathways were significant but the model fit was decent (R^2^ = 0.20; RMSEA = 0.07; CFI = 0.91; see Figure 1).

The intrinsic and integrated pathways were, however, borderline significant (at *p* < 0.10) and given the high levels of covariance between the factors, the non-significance of the pathways is likely due to suppressed covariance [47]. Put differently, the factors were unable to individually significantly predict physical activity because there was little unique variance available. Table 1 shows that each factor by itself was significantly associated with physical activity; see Supplementary Materials for *p*-value indicators.

**Figure 2:**
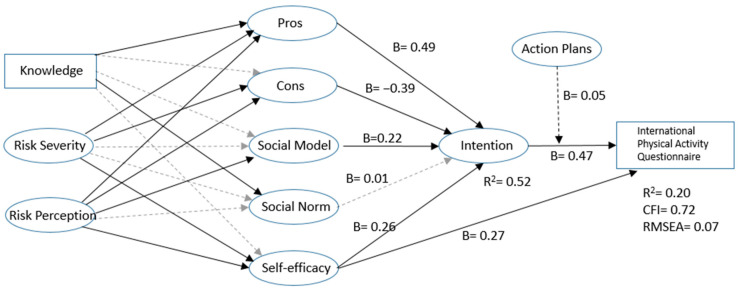
Unstandardized regression coefficients for ICM.

Second, the basic ICM model was tested. IPAQ scores at 3-month follow-up were regressed on intention and action planning. Action planning was regressed on intention and intention was regressed on pros, cons, social norms, social modelling and self-efficacy. These factors were then regressed on perceived risk, risk severity and knowledge. The model fit was mediocre at best (R^2^ = 0.19; RMSEA = 0.07; CFI = 0.73; see Figure 2).

Furthermore, intention was significantly predicted by pros, cons, social modelling and self-efficacy, with the greatest effect size was attributed to the pros. Action planning had a borderline significant (*p* < 0.10) moderation effect on the association between intention and physical activity, indicating that more action planning facilitated the translation of intentions into behaviour. The attitudinal concepts of pros and cons were well predicted by the distal factors knowledge, risk severity and risk perception (R^2^= 0.50 for the pros and R^2^ = 0.73 for the cons). The social concepts on the other hand were not well predicted by the distal factors (R^2^ = 0.15 for social norm, and R^2^ = 0.13 for social modelling).

### 3.3. Integration of SDT and ICM

**Figure 3:**
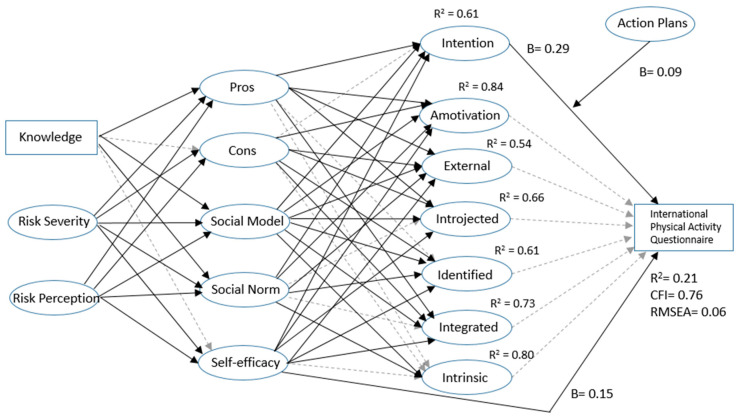
Unstandardized regression coefficients for the integrated model.

Both models were combined to assess whether the inclusion of additional factors would increase the model fit or explained variance. In the integrated model, we tried to stay close to the theoretical assumptions as formulated by the original authors. Furthermore, we assumed that ICM factors would account for a considerable amount of variance in SDT factors, which would subsequently account for the variance in intentions/behaviour (see Figure 3).

The results showed that the amount of explained behavioural variance (R^2^ = 21%) was similar to the results from the single SDT model and single ICM model. The model fit was slightly, but insignificantly, higher than the single ICM model and lower than the single SDT model.

The integrated model also showed that intention and self-efficacy were significant predictors of physical activity. In addition, the moderation effect of action plans on the association between intention and physical activity was significant, implying that intentions have a stronger effect on behaviour when action planning is high. Factors from SDT, however, were not significantly associated with behaviour. Furthermore, the explained variance of the cognitive factors was high (all > 54%).

## 4. Discussion

To aid theory in explaining and predicting behaviour, as well as ensure the effective planning of health-promoting programs, this study tested and integrated SDT and ICM for explaining physical activity behaviour using a longitudinal design. First, we assessed the model fit of ICM and SDT. The results showed that SDT fitted the data adequately. However, none of the factors could individually predict physical activity significantly, explained by the little unique variance available. We then tested the ICM model, with a similar, albeit slightly lower model fit as the STD. This indicates that both theories have a similar prediction power of physical activity. However, the ICM revealed specific pathways with attitude (pros), attitude (cons), social modelling and self-efficacy significantly predicting intention. Albeit with only borderline significance, the model also showed that action planning may facilitate intentions to actual behaviours. Moreover, the distal factors (i.e., knowledge, risk severity and risk perception) predicted to a certain degree these motivational factors as well.

Second, we integrated SDT and ICM to understand whether such an integrated model would have a better model fit. However, the integration of STD and ICM did not yield a significantly better prediction model than the stand-alone models. The ICM constructs revealed the underlying mechanism to physical activity well—with intention and self-efficacy as significant predictors. The relation between intention and behaviour was moderated by more action planning. Intention was in turn predicted by the motivational ICM constructs. This was all consistent with the pathways stipulated by ICM [5]. In contrast, SDT constructs had no significant pathways to behaviour. The high inter-correlations may provide an explanation. The inter-correlations may be due to suppressed variance; however, given the lack of additional explained variance in physical activity, it is probably caused by not containing sufficient unique predictive power when combined with the ICM constructs (i.e., intention, action plans and self-efficacy). Findings also indicate that the motivational factors from SDT, and the ICM factor known as intention, were all well explained by the attitude (pros as well as cons), social modelling, social norms and self-efficacy.

These findings are divergent from previous attempts to compare and integrate TPB and SDT. Previous studies suggest that STD constructs tend to form attitudes and perceptions of control. For instance, Luqman and colleagues (2018) [26] argued that autonomous and controlled motivation served as determinants of attitudes, subjective norms and perceived behavioural control. However, their design was cross-sectional and a reversed model, where STD constructs are predicted by the cognitive factors (i.e., attitude, social influence, self-efficacy/perceived behaviour control), was not considered. Previous attempts to integrate these constructs are heavily based on correlational data, which implies that alternative models with good fit may also exist [7]. Indeed, our findings support that cognitive factors actually predict the motivational state of behaviour change (intention of ICM, and the several STD constructs). As self-determined motivation can be supported or hindered by other factors, such as social support [48], our integrated model suggests that attitude, social influences, and self-efficacy are determinants of—rather than the product of—motivation. This supports the postulated underlying mechanism of the ICM pathways to the several types (or quality) of motivation, and how this in return predicts behaviour.

Our findings have several implications. First, ICM and STD have similar prediction power. Second, the integration of the models did not enhance explained variance. Therefore, when theory is used in research to maximise the explained variance of behaviour, the integration of both models can be considered inferior as it is less parsimonious than the stand-alone models; especially the SDT model. However, the ICM and especially the integrated model do reveal the underlying mechanism to how awareness and attitude, social influence, and self-efficacy actually predict the motivational state and in turn behaviour. When the models were combined, the pathways as stipulated by ICM remain significant, whereas the SDT constructs lacked the unique predictive power to have significant pathways to behaviour. Thus, the ICM may provide more guidance when used as a planning model, for intervention development and evaluation planning. Furthermore, our findings are in contrast to many integration attempts with regards to the placement of the motivation in relation to attitude, social influence, and self-efficacy/perceived behavioural control. Our study supports an alternative model with an important role of action planning for the intention, which should be further explored in future longitudinal research. Conclusions from previous studies indicate that individuals form future beliefs about outcomes and control over health-related behaviours if they have self-determined motives [49]. The integrated model is theoretically interesting as it provides a rationale behind the antecedents of self-determined motivation, as well as the determinants of attitude, social influence, and self-efficacy/perceived behavioural control [5,49]. It is thus interesting to further explore to what extent the reversed model applies; whether the socio–cognitive beliefs help motivation thrive with regard to health-related behaviours.

This study is not without limitations. First, this study is not completely comparable to previous attempts to integrate STD with TPB constructs. Previous studies often used only two SDT constructs [7], the autonomous and controlled motivation constructs. However, this study included the six original constructs postulated by SDT to preserve the richness of the model. Second, due to the focus of our study, and our study population, findings may not be generalizable beyond the Netherlands and physical activity. Third, items based on ICM constructs were derived from previous studies but were not extensively validated. However, the CFA can be regarded as an indicator of construct validity, and face/content validity checks were conducted by five health promotion researchers at Maastricht University. Future studies are needed to further study the criterion and concurrent validity. Fourth, we only used two items to assess susceptibility and also two items to assess the severity of mental/physical disabilities, resulting in relatively low Cronbach’s alphas of 0.56 and 0.69, respectively. However, finding a lower alpha is common for short scales and 0.50 can still be regarded as acceptable [50]. Cronbach’s Alpha is strongly influenced by the number of included items. With two item scales, the regular target values of 0.80 and over are much more difficult to achieve. However, despite the relative lower reliability values, significant relationships were established in the models, reflecting strong effects. Future studies may include more items for risk perception to ensure high internal consistency. Fifth, even though the model fit was deemed acceptable, the CFI and RMSEA values were not optimal. Sixth, no formal validation studies concerning the BREQ-3 exist for the Dutch population. However, the translated items were piloted among a small sample of young adults (reflecting no issues) and expert consultation also revealed no concerns about the items. However, it is recommended to validate the scale for use in future research. Last, physical activity is a high-level behaviour consisting of several sub-behaviours, making it a difficult behaviour to explain by one model. Thus, future research may shed light on the associations between STD and ICM constructs using other behaviours.

## 5. Conclusions

Using a longitudinal design this study showed that both the ICM and SDT have an adequate and similar model fit to physical activity data in the Netherlands. When integrating autonomy constructs of SDT with ICM, the explained variance remained the same, suggesting no added value as prediction model. When integrated, the ICM pathways remained standing whereas the SDT pathways to behaviour became non-significant. However, the integrated model corroborates prior research that showed TPB and SDT to be complementary in the sense that it explains the underlying mechanism. This explains determinants to STD constructs, as well as attitude, social influences, and self-efficacy. However, future research is needed to confirm the placements of these constructs in the model, and to explore other behaviours as well. This study indicates that ICM and SDT are similar in their predictive value, but in terms of intervention mapping, ICM or the integrated model seem most useful as this reveals the stages and pathways to behaviour change.

## Figures and Tables

**Table 1:** Estimated correlations between the factors.

	1	2	3	4	5	6	7	8	9	10	11	12	13	14	15	16
1. Amotivation																
2. External	0.31															
3. Introjected	−0.26	0.29														
4. Identified	−0.64	-	0.53													
5. Integrated	−0.40	0.11	0.48	0.68												
6. Intrinsic	−0.53	−0.05	0.33	0.71	0.72											
7. Risk perception	0.22	−0.09	−0.30	−0.34	−0.24	−0.19										
8. Risk severity	0.32	0.11	−0.18	−0.30	−0.15	−0.19	0.28									
9. Knowledge	0.17	-	−0.15	−0.17	−0.09	−0.12	0.11	0.12								
10. Pros	−0.47	0.07	0.45	0.61	0.55	0.59	−0.33	−0.32	−0.15							
11. Cons	0.42	0.14	−0.06	−0.44	−0.47	−0.61	-	0.12	0.06	−0.37						
12. Social norms	−0.18	0.13	0.20	0.19	0.12	0.12	−0.12	−0.18	−0.11	0.22	−0.05					
13. Social modelling	−0.07	0.14	0.09	0.13	0.19	0.17	-	−0.10	−0.06	0.18	−0.15	0.66				
14. Self-efficacy	−0.26	-	0.12	0.36	0.50	0.52	-	-	-	0.31	−0.55	-	−0.11			
15. Intention	−0.41	-	0.27	0.53	0.55	0.58	−0.14	−0.13	−0.13	0.43	−0.47	0.09	0.18	−0.43		
16. Action planning	−0.10	0.34	0.38	0.30	0.32	0.25	−0.15	−0.09	−0.09	0.36	−0.07	0.15	0.15	−0.10	0.26	
17. Physical activity	−0.22	-	0.12	0.30	0.37	0.38	−0.08	−0.10	−0.08	0.26	−0.30	−0.07	−0.13	0.32	−0.41	0.12

Note: - indicates a non-significant correlation (*p* > 0.05).

## Supplementary Materials

The following are available online at https://www.mdpi.com/1660-4601/18/1/28/s1, Table S1: Estimated correlations between the factors with *p*-value indication.
