# Supplementary material for: Combining the Integrated-Change Model with Self-Determination Theory: Application in Physical Activity
Source: Int J Environ Res Public Health. 2020 Dec 23;18(1):28. doi: 10.3390/ijerph18010028 (PMC7793065; doi:10.3390/ijerph18010028)
Supplement: Supplementary file 1 [file ijerph-18-00028-s001.pdf]

Supplementary Table S1: Estimated correlations between the factors with p-value indication.

	1	2	3	4	5	6	7	8	9	10	11	12	13	14	15	16
1. Amotivation																
2. External	.31**															
3. Introjected	-.26**	.29**														
4. Identified	-.64**	-.03	.53**													
5. Integrated	-.40**	.11**	.48**	.68**												
6. Intrinsic	-.53**	-.05*	.33**	.71**	.72**											
7. Risk perception	.22**	-.09**	-.30**	-.34**	-.24**	-.19**										
8. Risk severity	.32**	.11**	-.18**	-.30**	-.15**	-.19**	.28**									
9. Knowledge	.17**	.03	-.15**	-.17**	-.09**	-.12**	.11**	.12**								
10. Pros	-.47**	.07**	.45**	.61**	.55**	.59**	-.33**	-.32**	-.15**							
11. Cons	.42**	.14**	-.06*	-.44**	-.47**	-.61**	.05	.12**	.06**	-.37**						
12. Social norms	-.18**	.13**	.20**	.19**	.12**	.12**	-.12**	-.18**	-.11**	.22**	-.05**					
13. Social modelling	-.07**	.14**	.09**	.13**	.19**	.17**	-.05	-.10**	-.06*	.18**	-.15**	.66**				
14. Self-efficacy	-.26**	-.01	.12**	.36**	.50**	.52**	.02	-.04	.00	.31**	-.55**	.01	-.11**			
15. Intention	-.41**	.02	.27**	.53**	.55**	.58**	-.14**	-.13**	-.13**	.43**	-.47**	.09**	.18**	-.43**		
16. Action planning	-.10**	.34**	.38**	.30**	.32**	.25**	-.15**	-.09**	-.09**	.36**	-.07**	.15**	.15**	-.10**	.26**	
17. Physical activity	-.22**	.01	.12**	.30**	.37**	.38**	-.08*	-.10**	-.08**	.26**	-.30**	-.07*	-.13**	.32**	-.41**	-.11**

Note: * = p < 0.05; ** = p < 0.01
